# Effect of group size on pecking-injury prevalence and severity in male fattening turkeys (*Meleagris gallopavo* f. dom.)

**DOI:** 10.1016/j.psj.2026.108025

**Published:** 2026-10-22

**Authors:** Jessica Raabe, Gurubaran Raveendran, Leon Grude, Martin Streitenberger, Kai Homeyer, Helen Louton, Helen Schomburg, Thomas Bartels

**Affiliations:** a Friedrich-Loeffler-Institut, Institute for Animal Welfare and Animal Husbandry, Am Föhrkamp 1, Neustadt a. Rbge., D-31535, Germany; b University of Rostock, Faculty of Agriculture, Civil Engineering, and Environment, Professorship of Animal Health and Animal Welfare, Justus-von-Liebig-Weg 6b, Rostock, D-18059, Germany; c University of Applied Sciences and Arts, Institute for Sensor and Automation Technology, Ricklinger Stadtweg 120, Hannover, D-30459, Germany; d LMU Munich, Faculty of Veterinary Medicine, Department of Veterinary Sciences, Chair of Animal Welfare, Ethology, Animal Hygiene, and Animal Husbandry, Veterinärstr. 13/R, München, D-80539, Germany

**Keywords:** Turkey, Animal welfare, Animal husbandry, Injurious pecking, Group size

## Abstract

Agonistic interactions between fattening turkeys in large flocks often result in injurious pecking. In wild turkeys, ranking fights within familiar sibling groups appear from the 8th week of life, but the attacked bird usually escapes from superior birds. We compared three group sizes of male B.U.T. 6 turkeys (group I: n ≤ 11, group II: n ≤ 34, and group III: n ≤ 45) for performance and prevalence of injurious pecking across three trials over a 20-week fattening period. Temperature and relative humidity were logged. Turkeys were weighed on the 1st, 43rd, and 141st day of life (DOL), feed consumption was recorded after each feeding phase, and birds were inspected twice daily; when pecking injuries occurred, their degree and position were documented. Live weights did not differ among the three group sizes, although feed consumption was lower in group I from feeding phase P2 onward than in groups II and III (P < 0.01). Prevalence of injurious pecking was analyzed for low-grade and for combined moderate- and high-grade injuries. For all severity grades, prevalence was lower in group I than in groups II and III. Prevalence of low-grade injuries differed between all groups (P < 0.0001) with age-group size and age-bird number interactions. Moderate and high-grade injuries differed between groups I and II, between I and III (P < 0.0001), without differences between groups II and III, whereas some age-related differences occurred (P < 0.001), with an age-bird number interaction. Age and number of birds per group along with stocking density and available space, appeared to influence the prevalence of pecking injuries. With increasing age, growth, maturity, and less available space per compartment, agonistic interactions between conspecifics rose, whereas the head region was affected more often than the body, wing, or tail regions.

## Introduction

In all sectors of commercial turkey farming, including organic production, ‘injurious pecking’ between conspecifics can occur with high prevalence (Leishman et al., 2022a; Haug et al., 2023). Injurious pecking is a serious animal welfare problem that affects animal health in various ways and can cause high economic losses (Marchewka et al., 2013; Leishman et al., 2022b). Its etiology is not yet fully understood, so no animal-friendly yet reliably effective measures are currently available to prevent or minimize it (Leishman et al., 2022a). Besides genotype, sex, age, and maturation, turkey behavior is influenced by housing and management factors, such as stocking density, space availability, environmental enrichment, lighting, and feeding (Busayi et al., 2006; Marchewka et al., 2013; Erasmus, 2017; Grün et al., 2021; Krautwald-Junghanns et al., 2021; Kiani, 2022; Krautwald-Junghanns and Sirovnik, 2022; Dong et al., 2023; Kirn et al., 2025). Pecking injuries are a major problem in conventional turkey farming but also occur under organic guidelines that use slow-growing genotypes, smaller flocks with lower stocking densities, environmental enrichment, and outdoor access (Olschewsky et al., 2021; Haug et al., 2023; Kirn et al., 2025). Alongside exploratory behavior, agonistic behavior also appears to play a role with increasing age (Bartels et al., 2020). Particularly during fights for dominance, beak attacks are directed at the head and its appendages and the upper neck and can cause severe injuries (Bartels et al., 2020).

It is therefore necessary to analyze how selected housing factors promote or inhibit injurious pecking. One factor that has received little attention is group size (Abdel-Kafy et al., 2026). Most existing literature varies stocking density while keeping high bird numbers (e.g., Martrenchar et al., 1999; Duggan et al., 2014; Marchewka et al., 2015; Kulke et al., 2022) and does not substantially reduce group size to provide more space (Noll et al., 1991). To the best of our knowledge, this is the first study to investigate the effects of different group sizes on the prevalence and severity of pecking injuries in male fattening turkeys kept in small flocks over a 20-week period, from stabling to depopulation.

## Materials and methods

All husbandry conditions and procedures involving animal handling and treatment complied with the German Animal Protection Law and its associated by-laws and voluntary agreements (VDP, 2013; TierSchNutztV, 2016; TierSchG, 2022). Local ethics committee approval was not required according to LAVES (Lower Saxony State Office for Consumer Protection and Food Safety) notice from February 19, 2024.

### Animals, housing, and management

Across three trials, 1,065 intact-beak male day-old poults of the British United Turkeys 6 (B.U.T. 6) strain were kept for 20 weeks at the Experimental Station of the Institute for Animal Welfare and Animal Husbandry (Celle, Germany). In each trial, 355 poults were allocated to 12 barn compartments (18 m^2^ floor space) littered with wood shavings. Three group sizes were tested in four compartments per trial. On day of life (DOL) 1, compartments held either 10 to 11 turkeys (group I, n_total_ = 125 birds), 33 to 34 (group II, n_total_ = 405), or 44 to 45 (group III, n_total_ = 535). Target stocking densities at depopulation followed the B.U.T. 6 performance objectives (Aviagen, 2026): ≤ 12 kg/m² (≤ 0.6 turkeys/m²) in group I, ≤ 40 kg/m² (≤ 1.9 turkeys/m²) in group II, and ≤ 53 kg/m² (≤ 2.5 turkeys/m²) in group III. Each compartment had two feeders (Imperator; Big Dutchman, Vechta, Germany) and two round drinkers (Jumbo 98; Big Dutchman, Vechta, Germany) accessible from all sides. Turkeys were fed a standard six-phase diet (Fa. Agravis Raiffeisen AG, Münster, Germany), with food and water provided *ad libitum* throughout. Feed consumption was determined after each of the six feeding phases, on days 14, 35, 63, 91, 119, and 140. Each compartment contained pecking blocks (Vilovoss Deutsche Vilomix Tierernährung GmbH, Neuenkirchen-Vörden, Germany) for enrichment. When injurious pecking increased (n ≥ 2 turkeys with fresh injuries per compartment per day), additional dispensers with wheat grains were provided as enrichment (Berk et al., 2017). As part of an environmental enrichment the turkeys were given wheat grains that were not left freely available, but were provided in specially designed feeders (round troughs with a capacity of 10 liters, fitted with side openings through which the birds could only pick up individual grains when pecking). Studies have shown that these feed dispensers are actively used from the second week of life until the end of the fattening phase and can therefore be regarded as an effective measure for reducing environmental deprivation in turkey fattening (Berk et al., 2017). Furthermore, supplementing the diet with whole wheat grains can support gastrointestinal function in turkeys (Zdunczyk et al., 2013). Wheat consumed was recorded and added to regular feed intake. Compartments were individually climate- and light-controlled and shielded from external light. All birds were weighed as day-old poults on DOL 1, on the 43rd DOL after rearing when fattening started and on the 141st DOL when fattening finished for depopulation and slaughter. The number of weighing intervals was reduced to the minimum necessary to avoid stressful situations for the turkeys. Compartments were inspected at least twice daily, usually between 7:00 am and 9:00 am and between 1:00 pm and 3:00 pm. Mortality and causes of death—particularly severe injurious pecking—and the numbers of turkeys with fresh skin injuries from pecking or scratching were recorded twice daily (Knierim et al., 2020). Injury position (head, body, wing, or tail) was also noted. Injuries were classified into three severity levels (low-grade, moderate, and high-grade) following the animal welfare indicators of Knierim et al. (2020). The classification criteria are listed in Table 1. Birds with low-grade injuries were returned to their pen; those with moderate injuries were removed to a separate compartment (sick bay) until the end of fattening, turkeys with high-grade injuries required culling or were found dead. All injuries were treated with a zinc oxide skin-protection spray. Low-grade pecking injuries were analyzed alone. Analysis for moderate and high-grade injuries were in combination, because both injury grades required separation from the group either due to death/culling, or with prospect of recovery only through separation with treatment. The documentation of injuries is merely a snapshot of the situation at a given moment and, based on Bartels et al. (2020), moderate injuries can worsen significantly in a very short time and result in high-grade injuries. Temperature (T) and relative humidity (RH) were recorded at animal level in each of the 12 compartments throughout fattening with a data logger (Testo 177-H1, 4-channel; ranges RH 0 to 100%, T −20 to +70°C internal and −40 to +120°C external; Testo SE & Co. KGaA, Titisee-Neustadt, Germany).

**Table 1:** Criteria used to classify pecking injuries into the severity levels “low”, “moderate” and “high”.

**Grade of injury**	**Classification criterion of injuries**	**Procedure**
low	head region: diameter < 1 cm body region: diameter < 2 cm superficial injuries with a width < 0.3 cm that do not extend into the muscles	Treatment with Zinc oxide ointment for wound covering
moderate	head region: diameter ≥ 1 cm body region: diameter ≥ 2 cm deep injuries with a width ≥ 0.3 cm that extend into the muscles	Transfer to sick bay
high	deep, penetrating injuries to the head and/or neck, reaching skull, and/or spine	Culling due to dire prognosis

### Barn illumination

Each compartment was illuminated by two full-spectrum LED lamps (Zeus LED, Big Dutchman, Vechta, Germany) and shielded from external daylight. Their emission spectra included visible light (daylight, 6,500 Kelvin) and UVA (i.e., full-spectrum lighting, λ = 350-780 nm). Intensity was delivered through three light channels (‘Sunlike,’ ‘Lime,’ and ‘Ultraviolet’), all modulated at f = 16 kHz. Following VDP (2013) voluntary agreements, light was provided for 22 h on the day of arrival, with two dark phases from 2:00 pm to 3:00 pm and 7:00 pm to 8:00 pm. The light-dark [L:D] rhythm changed from 21 L:3 D on day 2 to 16 L:8 D on day 7 through the end of fattening, with 20-minute twilight phases in the morning and evening. In the first week of life, illuminance was reduced by 20 lx every 48 h from 100 lx to a minimum of 20 lx, reached in all compartments by day 7. Illuminance was measured at the turkeys’ eye level by a portable luxmeter (MAVOLUX digital, Gossen Foto- und Lichtmesstechnik GmbH, Nürnberg, Germany; measuring range: 0.1 to 199,999 lx, with error margins of maximum ± 3 %).

### Statistical analyses

Statistical analyses used RStudio (version 2024.9.0.375) and R (version 4.4.2) for Windows. The following block design was used: The barn involved two opposing sides, treated as two separated blocks, one facing south and one facing north. Each of the two blocks consisted of six compartments, whereas every group size was tested in two compartments per block and trial. The three trials were conducted time-independently of one another and were not included in the statistical models. After each trial, group sizes were changed and every group size was tested in every compartment for the whole fattening period.

***Performance Parameters.*** For live weights, birds were weighed individually by compartment (experimental unit), the values were further averaged by compartment and group. Feed consumption was recorded by compartment (experimental unit) and converted to the amount consumed per bird and day. Repeatedly measured live weights and feed consumption were analyzed with generalized least squares (GLS) assuming a 1st-order autoregressive correlation structure within the group. For live weights, the model used live weight as the dependent variable and weighing time point, group size, and block as categorical factors. For feed consumption, the model used feed intake, including wheat grains offered, as the dependent variable and feeding phase, group size, and block as categorical factors. Pairwise group comparisons for live weights per weighing time point and for feed consumption per feeding phase used a Tukey HSD post hoc test (R package emmeans, version 1.11.1), with P-values adjusted according to Tukey. Results are presented in means and standard deviations (SD), a confidence level of 0.95 and significance level of α = 0.05 was used.

***Climate Parameters.*** T and RH were continuously recorded per compartment (experimental unit) and analyzed for a relationship with the prevalence of low-grade or moderate and high-grade injuries using Spearman´s rank correlation (rS). Prevalence and climate data were grouped by the number of turkeys per compartment on DOL 1 (groups I, II, or III) and by WOL.

***Prevalence of Injurious Pecking.*** The prevalence of injurious pecking was documented per compartment (experimental unit) and analyzed per group. To reflect the dynamic fluctuations of bird numbers per compartment, regrouping was performed with bird numbers ranging between n = 9 to 11, n = 25 to 34, or n = 35 to 45 turkeys. Prevalence was separately calculated for low-grade, or moderate and high-grade injuries, since turkeys with injuries were counted once per category and the documentation for both categories were independently from one another. Both categories were analyzed with generalized linear models (GLM), quasibinomial distribution was assumed to avoid dispersion, including logistic regression as link function, and the number of turkeys on DOL 1 as denominator/weights. The dependent variable being the proportion of pecking injuries (low-grade or moderate and high-grade combined as numerator) relative to the number of animals per compartment on the 1st DOL (denominator). Categorical factors were group, age (week of life; WOL), and block. To retain information about the actual number of turkeys per compartment on the respective day, this variable was included as a numerical factor. A *Χ*²-test (chi-square, Wald statistic, type II deviance analysis) was performed, and nonsignificant interactions were removed stepwise by backward elimination. Comparisons among groups and between groups per WOL used estimated marginal means (emmeans) and a Tukey HSD post hoc test (R package emmeans, version 1.11.1) with Bonferroni P-value and 95% confidence level adjustment. Probabilities and confidence intervals (CI) for pecking injury comparisons among groups and between groups per WOL are displayed in square brackets.

***Localization of Pecking Injuries.*** The body parts affected by injurious pecking (head, body, wing, tail) were recorded by compartment (experimental unit) and analyzed for differences between group sizes. Positions were categorized as head-region injuries or summarized for body, wing, or tail injuries. Injuries including multiple body parts were treated independently and were analyzed separately for both categories, either for the head region or for the body, wing or tail region, since the location of the pecking injury is determined by the perpetrator’s motivation (Bartels et al., 2020). GLM used the selected body part as the dependent variable and group, WOL, and block (categorical), and the actual number of turkeys on the respective day (numerical) as independent variables. A quasipoisson distribution was assumed with a logarithmic link function. A *Χ*²-test (chi-square, Wald statistic, type II deviance analysis) was performed, and nonsignificant interactions were removed stepwise by backward elimination. Where variables differed significantly, a Tukey HSD post hoc test (R package emmeans, version 1.11.1) on the response scale with Tukey P-value adjustment, a 95% confidence level and a significance level of α = 0.05 was applied. Results for differences in the localization of pecking injuries between groups or WOLs were presented in rates with CIs in square brackets.

## Results

### Performance parameters

All turkeys were weighed on the 1st, 43rd, and 141st DOL (Table 2). On arrival, poults averaged 61.5 g (SD 5.0 g). On DOL 43, group I weighed 2.6 kg (SD 0.3 kg), and groups II and III averaged 2.7 kg (SD 0.3 kg). At the end of fattening on DOL 141, groups I and II weighed 23.6 kg (SD 1.4 kg and SD 1.6 kg, respectively), and group III weighed 23.7 kg (SD 1.4 kg). Live weights did not differ among the three groups. A significant interaction was found between weighing date and block (P < 0.05).

**Table 2:** Live weights of the turkeys on the 1st, 43rd and 141st day of life in relation to the group size.

**Age [DOL]**	**Live weights [kg]**					
	**Group I**		**Group II**		**Group III**	
	**M**	**SD**	**M**	**SD**	**M**	**SD**
1st	0.062	0.005	0.062	0.005	0.061	0.005
43rd	2.6	0.3	2.7	0.3	2.7	0.3
141st	23.6	1.4	23.6	1.6	23.7	1.4

No significant differences in live weights of the turkeys between the group sizes per weighing date were found. DOL: Day of life; M: Means; SD: Standard deviation.

Feed consumption was recorded after each of the six feeding phases; any wheat grains provided for enrichment were added to regular intake. Feed intake by group size is shown in Table 3. Group I differed from group II (t(197) = −3.4, P = 0.003) and III (t(197) = −3.1, P = 0.007) across all six phases, whereas group II and III did not differ. In phase P1, intake was highest in group I at 0.029 kg/animal/day, but from phase P2, it was lower in group I (0.098 kg/animal/day, SD 0.013) than in group II and III (both 0.102 kg/animal/day). In phase P3, group I again consumed less (0.23 kg/animal/day) but had the highest SD (0.05 kg/animal/day), whereas group III, with the most birds, had consistently low SDs.

**Table 3:** Feed consumption [kg/turkey/day] of the turkeys relative to the group size per feeding phases P1-P6, including additional wheat offered.

		**Group I**		**Group II**		**Group III**	
**Feeding phase**	**DOL**	**M**	**SD**	**M**	**SD**	**M**	**SD**
P1	1-14	0.029^a^	0.004	0.026^b^	0.003	0.025^b^	0.003
P2	15-35	0.098^a^	0.013	0.102^b^	0.005	0.102^b^	0.006
P3	36-63	0.23^a^	0.05	0.26^b^	0.02	0.26^b^	0.02
P4	64-91	0.44^a^	0.02	0.48^b^	0.03	0.47^b^	0.02
P5	92-119	0.57^a^	0.04	0.62^b^	0.06	0.61^b^	0.03
P6	120-141	0.60^a^	0.05	0.63^b^	0.04	0.65^b^	0.04

Differences in feed consumption of the turkeys between the group sizes per feeding phase were found. Means of groups I, II and III that share the same letter per feeding phase, were not found to differ significantly according to Tukey-Test. DOL: Day of life; M: Means; SD: Standard deviation.

For the six feeding phases in total, birds in group I consumed 0.33 kg/animal/day (SD 0.03), compared to groups II and III, where turkeys had a usage of 0.35 kg/animal/day (SD 0.026 for group II and SD 0.02 for group III).

### Climate parameters

Temperature and RH in each of the twelve compartments were recorded with data loggers. The mean temperature was 19.2°C (SD 3.7) in group I and 19.3°C (SD 3.9) in groups II and III. Mean RH was 58.2% (SD 16.8) in group I and 59.0% (SD 16.2) in groups II and III. From WOL 1 to 20, the mean temperature decreased from 25.0 to 16.0°C in group I, from 25.4 to 16.3°C in group II, and from 25.1 to 15.8°C in group III. Conversely, the mean weekly RH increased from 48.0 to 63.4% in group I, from 45.5 to 65.4% in group II, and from 46.3 to 66.6% in group III. Low-grade injuries related negatively to temperature (rS = −0.26, P < 0.05) and positively to RH (rS = 0.33, P < 0.01). Numbers of separated turkeys with moderate and high-grade injuries correlated negatively with temperature (rS = −0.56, P < 0.001) and positively with RH (rS = 0.5, P < 0.001).

### Prevalence of injurious pecking

Prevalence of injurious pecking was analyzed separately for low-grade and for combined moderate- and high-grade injuries (Table 4).

**Table 4:** Number of turkeys stabled at the 1st day of life (DOL) and depopulated at 141st DOL, turkeys removed from barn compartments as result of injurious pecking and turkeys removed without involvement of conspecifics.

**Group**	**Trial**	**Total number of turkeys in four compartment/trial**		**Total number of turkeys removed from four compartments/trial**			
				**as result of injurious pecking**		**without involvement of conspecifics**	
		**stabling** **[n]**	**depopulation** **[n]**	**transfer to sick bay** **[n; %]**	**killed by conspecifics or culled** **[n; %]**	**transfer to sick bay** **[n; %]**	**culled or dead** **[n; %]**
**I**	1	42	37	3; 7.1	0	0	2; 4.8
	2	43	40	0	0	2; 4.7	1; 2.3
	3	40	37	1; 2.5	0	0	2; 5.0
**II**	1	135	112	20; 14.8	1; 0.7	1; 0.7	1; 0.7
	2	134	106	25; 18.7	1; 0.8	1; 0.8	1; 0.8
	3	136	111	18; 13.2	3; 2.2	2; 1.5	2; 1.5
**III**	1	178	143	23; 12.9	7; 3.9	3; 1.7	2; 1.1
	2	178	145	27; 15.2	3; 1.7	0	3; 1.7
	3	179	143	22; 12.3	6; 3.4	5; 2.8	3; 1.7

***Prevalence of low-grade injuries.*** Across the three trials, low-grade injuries occurred in n = 10 birds (8.0%) in group I, n = 94 (23.2%) in group II, and n = 198 (37.0%) in group III. In the three trials, six compartments that began with 44 to 45 turkeys on DOL 1 were reduced by separations to 31 to 34 animals, comparable to the flock sizes in group II. Prevalence differences were therefore analyzed with rearranged groups of 9 to 11 (group I), 25 to 34 (group II), or 35 to 45 (group III) birds (categorial), and the total number of turkeys per compartment (numeric) to retain information and reflect the dynamic fluctuations of bird numbers. Mean low-grade injury prevalence was 0.006 (SD 0.08) in group I, 0.057 (SD 0.25) in group II, and 0.12 (SD 0.52) in group III. Low-grade prevalence showed significant interactions between group size and WOL (*Χ²* (df = 38) = 115.9, P < 0.0001) and between WOL and the number of animals on the respective day per compartment (*Χ*² (df = 19) = 86.1, P < 0.0001). Estimated marginal means with a 95% CI between the groups on the response scale showed a probability for the occurrence of low-grade pecking injuries of 2.8 × 10^-5^ [−6.1 × 10^-5^, 0.0001] for group I, significantly lower to group II with a probability of 5.5 × 10^-2^ [4.7 × 10^-2^, 0.06], compared to group III with highest probability among the groups with 3.0 × 10^-1^ [1.2 × 10^-1^, 0.5]. Group-size comparisons based on estimated marginal means on the response scale per WOL revealed differences in WOL 1, 6, 8, 10, and 17-20. In WOL 1, differences were found between group I and II, as well as between group I and III, but not between II and III. In WOL 6, 8, and 10, groups I and II differed from group III, without differences between groups I and II. In WOL 17-20, group I and II differed, but group I and III as well as group II and III did not. Table 5 presents the probabilities with CIs for the WOLs where differences occurred between the groups. Figure 1 shows turkeys with low-grade injuries per WOL by group size. In group I, the first low-grade injury appeared in WOL 10; seven injured turkeys were recorded across WOL 15-17 and two in WOL 19. In group II, low-grade injuries occurred in all WOLs except WOL 3. Prevalence was highest from WOL 14 on, with a weekly minimum of five injured turkeys (weeks 18 and 20) and a maximum of 12 (weeks 15 and 17). Low-grade injuries were consistently present in group III: a minimum of one case in WOL 5 and three in WOL 2, with five or more birds per week for the rest of fattening and a maximum of 24 in WOL 10. Between WOL 6-11, documented low-grade injuries totaled 10 or more per week.

**Table 5:** Age-dependent probabilities of low-grade pecking injuries with significant differences between group sizes.

**Probabilities for the occurrence of low-grade pecking injuries**					
**Age [WOL]**	**Group I** **Probability [95% CI]**	**Group II** **Probability [95% CI]**		**Group III** **Probability [95% CI]**	
1	< 0.001 [0.0, 0.0]		9.6 × 10^-1^ [8.1 × 10^-1^, 1.1]		> 0.99 [1.0, 1.0]
6	< 0.001 [0.0, 0.0]		1.1 × 10^-2^ [−1.8 × 10^-3^, 2.4 × 10^-2^]		3.9 × 10^-1^ [3.5 × 10^-2^,7.5 × 10^-1^]
8	< 0.001 [0.0, 0.0]		1.3 × 10^-2^ [−8.3 × 10^-3^, 3.5 × 10^-2^]		5.8 × 10^-1^ [6.9 × 10^-2^, 1.1]
10	8.0 × 10^-7^ [−1.8 × 10^-6^, 3.4 × 10^-6^]		6.0 × 10^-3^ [−4.2 × 10^-3^, 1.6 × 10^-2^]		5.8 × 10^-1^ [0.2, 1.0]
17	2.1 × 10^-4^ [−7.1 × 10^-4^, 1.1 × 10^-3^]		5.0 × 10^-3^ [1.4 × 10^-3^, 8.5 × 10^-3^]		7.5 × 10^-3^ [−1.1 × 10^-2^, 2.6 × 10^-2^]
18	< 0.001 [−7.0 × 10^-7^, 7.0 × 10^-7^]		2.3 × 10^-3^ [2.9 × 10^-4^, 4.4 × 10^-3^]		2.3 × 10^-3^ [−4.6 × 10^-3^, 9.2 × 10^-3^]
19	3.6 × 10^-5^ [−1.4 × 10^-4^, 2.16 × 10^-4^]		2.7 × 10^-3^ [7.2 × 10^-4^, 4.7 × 10^-3^]		2.1 × 10^-2^ [−3.6 × 10^-2^, 7.7 × 10^-2^]
20	< 0.001 [−4.0 × 10^-7^, 4.0 × 10^-7^]		1.9 × 10^-3^ [3.3 × 10^-4^, 3.6 × 10^-3^]		3.6 × 10^-3^ [−7.5 × 10^-3^, 1.5 × 10^-2^]

WOL: Week of life; CI: Confidence interval.

**Fig. 1:**
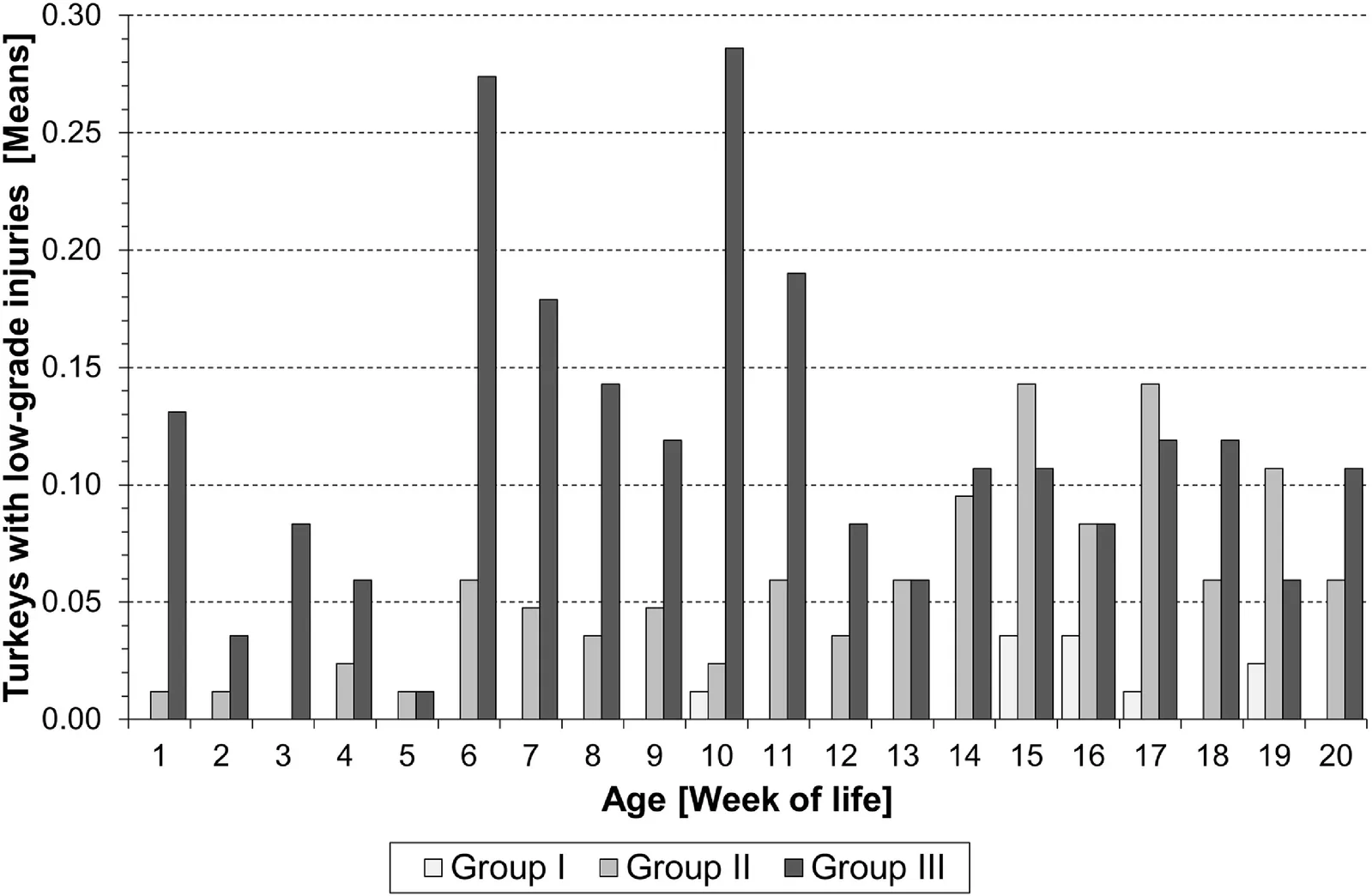
Turkeys with low-grade injuries, broken down by age, in the three study groups. The results are presented as mean values. Data on the number of turkeys per group and per trial run on the day of placement and at the end of the trial are listed in Table 4.

***Prevalence of moderate or high-grade injuries.*** Animals with moderate injuries (separated into a sick bay) or high-grade injuries (found dead or requiring culling) had the lowest incidence in group I, with four individuals (3.2%). In group II, 68 turkeys (16.8%) were separated, and in group III, 89 (16.6%) left the experimental compartment. For moderate and high-grade injuries, the main effects of WOL (*Χ*² (df = 19) = 45.5, P < 0.001), group size (*Χ*² (df = 2) = 52.0, P < 0.0001), and total number of turkeys on the respective day (*Χ*² (df = 1) = 23.4, P < 0.0001) were significant, with a WOL-by-number interaction (*Χ*² (df = 19) = 31.1, P < 0.05). Mean numbers and estimated marginal means on the response scale with a 95% CI of separated birds differed between group I (0.002, SD 0.05) with a probability of 7.3 × 10^-6^ [−4.9 × 10^-6^, 2.0 × 10^-5^] and group II (0.044, SD 0.2) with a probability of 1.9 × 10^-3^ [1.3 × 10^-3^, 2.4 × 10^-3^] and between groups I and III (0.05, SD 0.3) showing a probability of 7.1 × 10^-3^ [1.4 × 10^-3^, 1.3 × 10^-2^], with no difference between groups II and III. In group I, two turkeys were separated in each of WOL 16 and 19. In group II, no birds were separated during WOL 1-5, and single cases occurred in WOL 6 and 9. From WOL 11 on, birds were separated weekly, from a minimum of one (WOL 12 and 13) to a maximum of 13 (WOL 17). In group III, three turkeys were separated during the 1st WOL. Prevalence rose from WOL 11 on (four cases) and from WOL 13 on, with five or more birds separated weekly. From WOL 17 on, ten or more birds per week were affected. Table 6 shows the probabilities for severe injuries with CIs for those WOLs where differences occurred between the groups. Between-group differences per WOL appeared in WOL 14 and 15: group I (no birds removed) differed from group II (7 separated in WOL 14 and 10 in WOL 15) but not from group III (5 in WOL 14 and 8 in WOL 15), and groups II and III did not differ. In WOL 16 to 18, group I (two birds removed in WOL 16) differed from both group II (27 removed) and group III (31 removed), whereas groups II and III did not differ. In WOL 19, all three groups differed, with two turkeys separated in group I and 11 in each of groups II and III. In WOL 20, group I (no birds) differed from group II (4 separated) but not from group III (10), and groups II and III did not differ.

**Table 6:** Age-dependent probabilities of moderate and high-grade pecking injuries with significant differences between group sizes.

**Probabilities for the occurrence of moderate or high-grade pecking injuries**			
**Age [WOL]**	**Group I** **Probability [95% CI]**	**Group II** **Probability [95% CI]**	**Group III** **Probability [95% CI]**
14	1.6 × 10^-5^ [−1.2 × 10^-5^, 4.4 × 10^-5^]	4.1 × 10^-3^ [5.0 × 10^-4^, 7.6 × 10^-3^]	1.6 × 10^-2^ [2.9 × 10^-3^, 3.4 × 10^-2^]
15	2.3 × 10^-5^ [−1.6 × 10^-5^, 6.3 × 10^-5^]	5.9 × 10^-3^ [1.9 × 10^-3^, 9.9 × 10^-3^]	2.2 × 10^-2^ [−5.1 × 10^-4^, 4.5 × 10^-2^]
16	2.3 × 10^-5^ [−1.5 × 10^-5^, 6.0 × 10^-5^]	5.8 × 10^-3^ [2.5 × 10^-3^, 9.1 × 10^-3^]	2.2 × 10^-2^ [1.6 × 10^-3^, 4.2 × 10^-2^]
17	2.0 × 10^-5^ [−1.7 × 10^-5^, 5.7 × 10^-5^]	5.2 × 10^-3^ [2.4 × 10^-3^, 7.9 × 10^-3^]	2.0 × 10^-2^ [2.6 × 10^-3^, 3.7 × 10^-2^]
18	1.2 × 10^-5^ [−1.1 × 10^-5^, 3.4 × 10^-5^]	3.0 × 10^-3^ [1.1 × 10^-3^, 4.9 × 10^-3^]	1.2 × 10^-2^ [9.0 × 10^-4^, 2.2 × 10^-2^]
19	2.0 × 10^-5^ [−1.7 × 10^-5^, 5.7 × 10^-5^]	5.2 × 10^-3^ [3.1 × 10^-3^, 7.3 × 10^-3^]	2.0 × 10^-2^ [4.5 × 10^-3^, 3.5 × 10^-2^]
20	5.3 × 10^-6^ [−5.3 × 10^-6^, 1.6 × 10^-5^]	1.3 × 10^-3^ [2.5 × 10^-4^, 2.4 × 10^-3^]	5.2 × 10^-3^ [−3.3 × 10^-4^, 1.1 × 10^-2^]

WOL: Week of life; CI: Confidence interval.

***Localization of Pecking Injuries.*** Injury positions were categorized as head, body, wing, or tail. Of 186 documented injuries, 132 (71.0%) were in the head region, 17 (9.1%) on the body, 35 (18.8%) on the wings, and two (1.1%) on the tail. In 45 cases, more than one position was affected.

Most head-region injuries occurred from WOL 10 on (n = 128): four (3.1%) in group I, 63 (49.2%) in group II, and 61 (47.7%) in group III. On the body, two birds (11.8%) were in group I, six (35.3%) in group II, and nine (52.9%) in group III. One bird (2.9%) had wing injuries in group I, 16 (45.7%) in group II, and 18 (51.4%) in group III; both injured tails were in group III. Head-region prevalence and rates of estimated marginal means differed between groups (*Χ*² (df = 2) = 61.0, P < 0.0001): group I (0.002, SD 0.05) with a rate of 0.0002 [−6.3 × 10^-5^, 5.0 × 10^-4^] versus group II (0.04, SD 0.2) with a rate of 0.05 [4.0 × 10^-2^, 6.0 × 10^-2^] and P < 0.0001, group I versus group III (0.04, SD 0.2) with a rate of 0.2 [5.5 × 10^-2^, 0.3], with P = 0.012, and group II versus III with P = 0.08. Head injury means also differed between WOLs (*Χ*² (df = 19) = 64.3, P < 0.0001): Differences based on estimated marginal means were found between WOL 19 with a rate of 0.2 [0.1, 0.4] versus WOLs 1 [−6.3 × 10^-6^, 6.3 × 10^-6^], 2 [−6.1 × 10^-6^, 6.1 × 10^-6^], 3 [−6.0 × 10^-6^, 6.0 × 10^-6^], 4 [−5.9 × 10^-6^, 5.9 × 10^-6^], 5 [−5.9 × 10^-6^, 5.9 × 10^-6^], 8 [−5.6 × 10^-6^, 5.6 × 10^-6^], and 9 [−5.6 × 10^-6^, 5.6 × 10^-6^]. Rates for WOLs 1-5, 8, and 9 were all < 0.001 with P < 0.05.

For body, wing, and tail injuries, prevalence differed between groups I and II (*Χ*² (df = 2) = 17.9, P = 0.0001): group I (0.002, SD = 0.06) with a rate of 9.0 × 10^-5^ [−1.0 × 10^-4^, 3.0 × 10^-4^] versus group II (0.014, SD = 0.1) with a rate of 0.02 [0.01, 0.03] and P = 0.0005. No significant differences were found between group I versus group III (0.02, SD = 0.2) with a rate of 0.1 [−0.05, 0.3] and between groups II versus III. For the body, wing, and tail regions, no differences were found between WOLs.

## Discussion

### Performance parameters

Live weights did not differ among the three groups at the end of the fattening period, whereas mean feed consumption in groups II and III was higher from feeding phase P2 on. The turkeys’ live weights slightly exceeded the B.U.T. 6 performance objectives of 21.5 kg on DOL 140 (Aviagen, 2026) versus 23.65 kg on DOL 141, although weight on DOL 1 was slightly lower at 61.5 g than the 62.8 to 63.3 g reported by Jankowski et al. (2015) for poults of the same strain. A comparable excess of about 9% was reported by Raabe et al. (2024) in small groups of 45 turkeys under similar conditions, where live weights were approximately 8% above the breeder’s data. In contrast, Martrenchar et al. (1999) observed increasing body weight with greater floor space, whereas feeding duration was unaffected. Jankowski et al. (2015) found a higher final body weight and in some cases, a significantly higher daily feed intake, for turkeys in groups of 28 versus 34 birds (both at 10 m²); however, the latter were kept at a higher temperature that appeared to reduce feed consumption and thus weight gain. Raabe et al. (2024) reported similar feed consumption in groups of 33 to 34 and 44 to 45 turkeys, and the higher consumption in our larger groups suggests that birds may be more attracted to feed by conspecifics. Animals may also be disturbed more often by group mates with higher stocking density and less available space, as observed by Martrenchar et al. (1999), increasing foraging motivation or competition for food (Irfan et al., 2016) without affecting final live weights.

### Climate parameters

Although low-grade pecking injuries tended to relate to RH, and moderate including high-grade injuries correlated negatively with T and positively with RH, direct conclusions of environmental variables on the prevalence of pecking injuries should be avoided. Both T and RH changed systematically for all groups with bird age and were under control. Higher temperatures were maintained early in the fattening period, when injurious pecking was low anyway, and prevalence rose with the age of the turkeys, along with falling T and rising RH towards depopulation. Nevertheless, observations in laying hens showed an increase of skin injuries at higher temperatures (Liebers et al., 2019). However, González-Zapata et al. (2021) kept small flocks of 15 turkeys under heat stress throughout and found no aggressive behavior, possibly because the small group size, outdoor access, and daily recurring heat stress kept birds highly engaged in thermoregulation (González-Zapata et al., 2021; Sake et al., 2024). Mendes et al. (2020) described climate associations in turkeys aged 96 and 131 days; exposure to 26°C with elevated humidity of 76.2% was associated with more injurious pecking than exposure to higher temperatures. Le Bris (2005) and Jankowski et al. (2015) likewise found reduced display and more resting in B.U.T. 6 turkeys on pasture at higher temperatures. Although no direct effect of T and RH on the prevalence of pecking injuries can be presumed in regard of the current results, the monitoring of environmental variables is essential, to reduce experimental errors affecting the results (Farokhmoradi and Salari-Kakhk, 2025).

### Prevalence of injurious pecking

Low-grade injury prevalence showed significant interactions between group size/bird number and age. Low grade pecking injuries differed between the three groups, whereas separated birds with moderate or high-grade pecking injuries differed between groups I and II and between groups I and III, with no difference between groups II and III.

Injurious pecking is a multifactorial issue: sex, age, flock size, available space, and environmental factors all influence its occurrence (e.g., Dalton et al., 2013; Marchewka et al., 2013; Duggan et al., 2014; Irfan et al., 2016; Ferrante et al., 2019; Bartels et al., 2020; Schäfers et al., 2024). It can develop independently of the housing system in conventionally raised turkeys (Krautwald-Junghanns et al., 2011) and under organic conditions (Olschewsky et al., 2021; Haug et al., 2023). Its causes and origins in domesticated turkeys are not yet fully understood (Dalton et al., 2013, 2018; Duggan et al., 2014), and it may persist in modern husbandry as a legacy of wild ancestors (Leishman et al., 2022a). Agonistic behavior—fights for social hierarchy and dominance—is part of the complex social behavior of wild turkeys that intensely and frequently occurs during spring and fall flock consolidation (Watts, 1969) of dioeciously living groups up to 30 females and 2 to 6 males in spring (Watts, 1969; Krakauer, 2005), that merge into larger single-sex winter flocks during fall (Watts, 1969; Swearingin et al., 2010).

Injuries including multiple body parts were analyzed independently of one another and separately for the positions, since the location of the pecking injury is determined by the motivation of the perpetrator. During hierarchical disputes the head region was, in agreement with Bartels et al. (2020), affected far more with increasing age, than the other body parts, that were pecked due to redirected environmental or plumage pecking. This was also confirmed by Dalton et al. (2013), who states that head-region injuries mainly arise from increasing agonistic interactions and hierarchical disputes at the onset of maturity. For male flocks of 2,250 to 4,000 birds at DOL 122 to 138, Ferrante et al. (2019) reported higher injury means than the current study—0.3 (SD 0.01) for head/neck, 0.1 (SD 0.01) for back, and 0.5 (SD 0.05) for tail wounds. In Ferrante et al. (2019), stocking density was 2.7 to 4.1 males per m², versus 2.4 to 2.5 per m² in the current study. The higher injury rates in these large flocks suggest that the group size may have an influence on the development of agonistic behavior (Ferrante et al., 2019).

Buchwalder and Huber-Eicher (2003) showed that groups of four male domesticated turkeys form a stable unit that distinguishes group members from non-members, with agonistic interactions being more frequent between unfamiliar groups than within familiar members. They also observed that introduced, unfamiliar males were keen to join a stable group of five toms, indicating that hybrid lines also aspire social dominance systems (Buchwalder and Huber-Eicher, 2004). Agonistic interactions toward an unfamiliar conspecific were more frequent in small groups of six than in larger groups of 30 males, and individual recognition was presumably limited at 30 birds (Buchwalder and Huber-Eicher, 2005). Even larger groups, however, appear able to recognize conspecifics from other groups: Olschewsky et al. (2021) observed groups of 50 free-range turkeys and reported three birds being pecked after escaping into a group of unfamiliar conspecifics.

Unlike Buchwalder and Huber-Eicher (2003, 2004, 2005), we did not confront familiar groups with unfamiliar turkeys. To the best of our knowledge, this is the first study to focus on how fattening turkeys develop in small group sizes across the entire fattening period. Group sizes close to the natural clutch size of 9-11 birds (Tyl et al., 2020; Johnson et al., 2022) were tested against groups of initially 33 to 34 and 44 to 45 turkeys from DOL 1 to 141. These group sizes correspond to stocking densities that are either recommended in Germany for male turkeys (group II: 40 kg/m²) or standard on turkey farms (group III: 53 kg/m²) (Krautwald-Junghanns and Sirovnik, 2022). However, small flocks of up to 45 birds depart from conventional practice and therefore should be regarded as part of a fundamental research setting. This approach confounds group size with stocking density and available space, but still gives insight into the behavior of fattening turkeys in small flocks (Leone and Estevez, 2008; Duggan et al., 2014). Several studies analyzed behavior and injurious pecking in compartments with less space at conventional stocking densities (e.g., Wu and Hocking, 2011; Berk et al., 2017; Bartels et al., 2020; Lindenwald et al., 2021; Struthers et al., 2022; Dong et al., 2023; Haug et al., 2023; Raabe et al., 2024), but how far these results transfer to agricultural practice remains to be seen.

In the present study, higher rates for low-grade or moderate- and high-grade pecking injuries were found in groups with higher bird numbers, along with higher stocking densities and less available space, but pecking injuries in the groups cannot be interpreted separately from stocking densities and space allowance. Moreover, stocking densities changed daily until depopulation with birds’ growth and separation of turkeys from compartments. Therefore, the group size with bird number range was used to reflect these dynamic fluctuations. The first separations from group III were in WOL 1 and 3, whereas in group II the 1st turkey was removed in WOL 6. The highest numbers of moderate or high-grade injuries occurred in group III, which decreased from 44 to 31 birds: the 1st three birds were separated on DOL 7, and the 13th was separated on DOL 131. Only four birds were separated from group I; although playful disputes may have occurred, no other pecking injuries were recorded aside from these. These results support the finding of Buchwalder and Huber-Eicher (2005) that fattening turkeys in small flocks may individually identify group members, a potential requirement to establish stable social hierarchies through ranking fights. Pecking injuries also occurred in group I, but far less and low-grade injuries appeared later than in groups II and III. Buchwalder and Huber-Eicher (2003) also noted aggressive head pecking between familiar members, but less often than between non-members. Healy (1992) found wild turkey sibling groups begin fighting for dominance from the 8th WOL on, comparable to group I in the present study and to Le Bris (2005), who observed display behavior begin in the rearing phase, rising to 13% by WOL 8. From this age, agonistic interactions and dominance fights became more frequent, also shown by Bartels et al. (2020). In contrast, Abdel-Kafy et al. (2026) observed higher agonistic-interaction rates in mixed-sex flocks at WOL 4 to 8 than later at WOL 10 and 12.

It can be supposed that a stable social order can more likely be established in the smallest groups with most available space for the victim to remove from opponents. In compartments with 9 to 11 turkeys, fewer conspecifics may have been drawn into the chase— like observed by Le Bris (2005) during rearing and by Bartels et al. (2020) in groups of 45, where increasing numbers of birds intervened in fights. In a comparable study of small flocks of initially 45 males, Raabe et al. (2024) separated 19.9% of birds for severe injuries, similar to the 16.6% removed from group III here. Abdel-Kafy et al. (2026) recorded turkeys in groups of 25 or 75 (0.5 birds/m²) and observed more frequent aggression in the large group, indicating that the bird number rather than the stocking density or available space drives agonistic behavior and hierarchical encounters. Concomitant with this assumption, a study by Kulke et al. (2022) showed that a reduction of the stocking density in flocks of 40 kg/m² (1,135 turkey poults) compared to 58 kg/m² (1,645 turkey poults) was not significantly decreasing the prevalence of pecking injuries, although space allowance was greater in the smaller flock.

Whether a hierarchical dispute ends in a low-grade or severe injury is determined by several contributing factors and not only by the number of turkeys per group, but is also influenced by the age, respectively, and thus maturity of the males. In this study, the tested group sizes, however, are inextricably linked with available space and stocking density, but our data nevertheless indicates that possible mechanisms like age, together with growth and space, influences the incidence of agonistic interactions, with pecking injuries rising until slaughter—also reported by Olschewsky et al. (2021) and Schäfers et al. (2024). Ranking fights and injurious pecking presumably bear a higher risk to occur in male flocks with the onset of maturity plus high bird numbers of several thousands, typical for commercial farming (Martrenchar et al., 1999; Buchwalder and Huber-Eicher, 2005; Irfan et al., 2016; Ferrante et al., 2019). The stable linear hierarchy that wild turkey males establish at a small scale to ensure dominance, flock stability, and survival may be problematic to be formed in large flocks, conceivably in consequence of missing familiarity and an impeded identification between conspecifics, high stocking densities and restricted space (Watts, 1969; Marchewka et al., 2013, (Marchewka et al., 2020)). Results from small experimental groups therefore cannot ultimately be compared and transferred to commercial farms, but are valuable to gain knowledge on turkey behavior in a small scale (Abdel-Kafy et al., 2026). Unlike the wide habitats of wild turkeys, conventional husbandry offers limited space, whereas the EFSA AHAW Panel (2026) calculated space allowances of 0.74 m² per tom at 16 kg and 0.82 m² per tom at 25 kg target body weight for birds to perform relevant behavior—more space per bird than usual in conventional husbandry.

Lacking reliably effective measures to prevent injurious pecking, conventional farming commonly uses non-curative surgical interventions to reduce the risk of injury, though these do not prevent it entirely (Krautwald-Junghanns et al., 2011; Kulke et al., 2022). Depending on national law, infrared beak treatment (‘beak trimming’) is routinely performed on day-old poults at the hatchery (Struthers et al., 2022; Alfirevich et al., 2025). Some countries also perform ‘de-snooding’—surgical removal of the processus frontalis (‘snood’), a raisable fleshy protrusion on the forehead—to prevent future pecking damage (Alfirevich et al., 2025). Whether such mutilations are permitted under national animal welfare law varies among EU member states. From a welfare perspective, however, both injurious pecking and the prophylactic mutilation of the upper beak tip and the snood are viewed critically (EFSA AHAW Panel, 2026). In principle, it is not the animals that should be adapted to commercial husbandry environments; rather, farming conditions should be adjusted to the needs of the birds (Irfan et al., 2016).

### Future directions

Our results show that keeping intact-beaked turkey flocks at small-scale, accompanied by a substantially lower stocking density along with an increased space allowance, benefits bird health and welfare and, presumably, economics, through reduced injury rates and losses. But, raising fattening turkeys in small groups with low bird numbers instead of large flocks will undoubtedly be economically challenging for livestock farmers. Nonetheless, this study confirms previous observations that bird age and the resulting sexual maturity in males likewise affect the frequency of severe pecking injuries, particularly to the head (Bartels et al., 2020). Housing and management measures and mechanisms for control or to delay sexual maturation could therefore additionally help reduce hierarchical disputes and pecking injuries.

The low-stimulus environment of commercial husbandry contradicts the natural habitats of wild turkeys (Watts, 1969; Kulke et al., 2022; Johnson et al., 2022), whereas structural elements and flexible illumination could create more structured conditions and improve animal housing. Artificial light and natural daylight strongly influence animal behavior and regulate physiological processes (Vehse and Ellendorff, 2001; Yahav et al., 2000). As days grew longer, Vermette et al. (2016) observed a linear increase in inactive resting and a linear decrease in exploratory behavior and feather pecking. German recommendations (VDP, 2013) suggest 16 h light and 8 h darkness, similar to late spring and summer light conditions, where wild turkeys form smaller groups and agonistic ranking fights for dominance peak (Watts, 1969). Classen et al. (1994) reared 1,620 male turkey chicks for 188 days and found that birds on the intermittent lighting programs walked better, were more active and injurious pecking was more prevalent than under continuous light, suggesting that lighting-based control of plasma testosterone relevantly influences bird behavior and may substantially reduce agonistic interactions even with increasing age of the turkeys. Another approach to minimize agonistic behavior could therefore be to adjust lighting duration—particularly day length—to mimic fall or winter conditions, allowing the turkeys’ hormonal status to adapt to a more inactive behavior. However, this must first be scientifically validated.

Keeping male fattening turkeys in small flocks along with a lower stocking density and increased space, provided important insight into bird behavior in this study. The present results indicate, that the common practice of housing several thousand individuals together may conflict with the turkeys’ natural behavior and welfare needs. The turkeys observed here had no outdoor access, that can be relevant to enhance animal welfare and limit the prevalence of agonistic interactions, like in Irfan et al. (2016), who reported a higher aggression in confined turkeys than in free-range turkeys. Taken together, previous work indicates that wild turkey behavioral traits persist in commercial birds, so husbandry conditions should be more oriented to the turkeys’ requirements, that can, other than substantially reducing the bird number per flock, be the enrichment of the housing environment, the provision of structural elements and intensive flock observations were also suggested to reduce losses and improve welfare and health in conventional turkey fattening (e.g., Dalton et al., 2013; Marchewka et al., 2013; Duggan et al., 2014; Irfan et al., 2016; Ferrante et al., 2019; Bartels et al., 2020; Kulke et al., 2022; Schäfers et al., 2024). Overall, further research on how smaller flocks affect the development and characteristics of agonistic behavior in the conventional fattening turkey sector is warranted.

## Author contributions

**J. Raabe** performed substantive input into the design of the work, designed the experimental procedures, conducted the experiments and video analyses, analyzed the data, addressed their interpretation from the perspective of animal welfare and animal husbandry, wrote the initial manuscript draft and developed the revision of the reviewed manuscript. **G. Raveendran** developed the technical experimental design of the study, developed and monitored the technical setups, handled data curation and formal analyses, and critically revised the manuscript. **L. Grude** performed substantive input into the design of the work, designed the experimental procedures, assisted with the installation and monitoring of technical setups, data management, and data analysis, and critically revised the manuscript. **M. Streitenberger** conceived the study, interpreted the experimental results, and critically revised the manuscript. **K. Homeyer** designed the study, reviewed the scientific content, refined the interpretation of the results, and reviewed the draft critically for crucial intellectual content. **H. Louton** evaluated the experimental design and the veterinary significance of the results, integrated the key findings, and critically revised the manuscript for important intellectual content. **H. Schomburg** assessed the scientific interpretation of the results, verified the coherence of the results and conclusions, and critically revised the manuscript. **T. Bartels** conceived the study, established the scientific framework, evaluated the biological significance of the results, addressed their interpretation from the perspective of animal welfare and animal husbandry, and critically revised the initial manuscript draft and the revision of the submitted manuscript. All authors have reviewed and expressly approved the final version of the manuscript prior to submission as well as the second draft of the manuscript revised due to two anonymous reviewer expert opinions. They accept personal and public responsibility for all aspects of the work, including its accuracy and integrity, as well as the appropriate resolution of any questions concerning the work.

## Disclosures

The authors confirm that there are no conflicts of interest regarding any financial, personal, or other relationships with other people or organizations that could inappropriately influence or be perceived to influence this study.
